# Transcriptome analysis of *Deinagkistrodon acutus *venomous gland focusing on cellular structure and functional aspects using expressed sequence tags

**DOI:** 10.1186/1471-2164-7-152

**Published:** 2006-06-15

**Authors:** Bing Zhang, Qinghua Liu, Wei Yin, Xiaowei Zhang, Yijun Huang, Yingfeng Luo, Pengxin Qiu, Xingwen Su, Jun Yu, Songnian Hu, Guangmei Yan

**Affiliations:** 1 James D. Watson Institute of Genome Sciences, Zhejiang University, Hangzhou, China; 2 Beijing Institute of Genomics, Chinese Academy of Sciences, Beijing, China; 3 Department of Pharmacology, Zhongshan Medical School of Sun Yat-sen University, Guangzhou, China; 4 Department of Biochemistry, Zhongshan Medical School of Sun Yat-sen University, Guangzhou, China

## Abstract

**Background:**

The snake venom gland is a specialized organ, which synthesizes and secretes the complex and abundant toxin proteins. Though gene expression in the snake venom gland has been extensively studied, the focus has been on the components of the venom. As far as the molecular mechanism of toxin secretion and metabolism is concerned, we still knew a little. Therefore, a fundamental question being arisen is what genes are expressed in the snake venom glands besides many toxin components?

**Results:**

To examine extensively the transcripts expressed in the venom gland of *Deinagkistrodon acutus *and unveil the potential of its products on cellular structure and functional aspects, we generated 8696 expressed sequence tags (ESTs) from a non-normalized cDNA library. All ESTs were clustered into 3416 clusters, of which 40.16% of total ESTs belong to recognized toxin-coding sequences; 39.85% are similar to cellular transcripts; and 20.00% have no significant similarity to any known sequences. By analyzing cellular functional transcripts, we found high expression of some venom related genes and gland-specific genes, such as calglandulin EF-hand protein gene and protein disulfide isomerase gene. The transcripts of creatine kinase and NADH dehydrogenase were also identified at high level. Moreover, abundant cellular structural proteins similar to mammalian muscle tissues were also identified. The phylogenetic analysis of two snake venom toxin families of group III metalloproteinase and serine protease in suborder *Colubroidea *showed an early single recruitment event in the viperids evolutionary process.

**Conclusion:**

Gene cataloguing and profiling of the venom gland of *Deinagkistrodon acutus *is an essential requisite to provide molecular reagents for functional genomic studies needed for elucidating mechanisms of action of toxins and surveying physiological events taking place in the very specialized secretory tissue. So this study provides a first global view of the genetic programs for the venom gland of *Deinagkistrodon acutus *described so far and an insight into molecular mechanism of toxin secreting.

All sequences data reported in this paper have been submitted into the public database [GenBank: DV556511-DV565206].

## Background

Venomous snakes possess one of the most sophisticated integrated weapons system in the natural world. It has been hypothesized that the snake venom gland itself evolved in the mouth region as a consequence of an evolutionary change in the pancreatic trait, and consequently, some of the toxins should show strong affinities to pancreatic proteins [1]. Recent studies suggested that snake venom components such as phospholipase A2 have evolved in an accelerated manner to acquire their diverse physiological activities [2]. Most studies of snake venom glands have focused almost exclusively on the components of venom. Therefore, a fundamental question being arisen is what genes are expressed in the snake venom glands besides many toxin components? To obtain a more comprehensive view of function of the venom gland, it is necessary for us to choose a proper model to study gene expression and toxin diversity in snake venom gland and perform comparative research with other species.

*Deinagkistrodon acutus *(*D. acutus*) is a specific snake in Southern China and many toxin components from the venom gland of *D. acutus *have been purified and characterized [3-6]. More studies for *D. acutus *venom gland will promote the application of toxin in medicine. To our best knowledge, there is little information about the *D. acutus *venom gland at the molecular level. This problem is compounded by the limited number of annotated *D. acutus *nucleotide sequences currently deposited in the public databases (<82). For this reason, *D. acutus *should be a good model for use in studying gene expression and organogenesis in snake venom gland. Analysis of expressed sequence tags (ESTs) is an efficient approach for gene discovery, expression profiling [7-9] and development of resources useful for functional genomics studies. For this purpose, we decided to adopt the strategy of large scale sequencing by constructing a cDNA library from the venom gland of *D. acutus*. Thus we should be able to find relevant genes and investigate their function after screening and expressing the target genes.

Knowledge of genes and proteins expressed in the various venom glands offers a potential resource to guide future investigations relevant to biology of venom gland and toxicology. For instance, proteins secreted from the venom gland of *D. acutus *represent a subset of candidates potentially involved in local and systemic effects as pain, edema, bleeding and muscle necrosis. Moreover, gene cataloguing and profiling of the venom gland of *D. acutus *is an essential requisite to provide molecular reagents for functional genomic studies needed for the elucidation mechanisms of action of toxins and the discovery of their antagonists [10]. On the other hand, it will allow the identification of cellular functional transcripts that may represent a general panorama of the physiological events of the venom glands, surveying gene expression from the very specialized secretory tissue.

## Results and discussion

### Overview of ESTs from the venom gland of *D. acutus*

**Figure 1:**
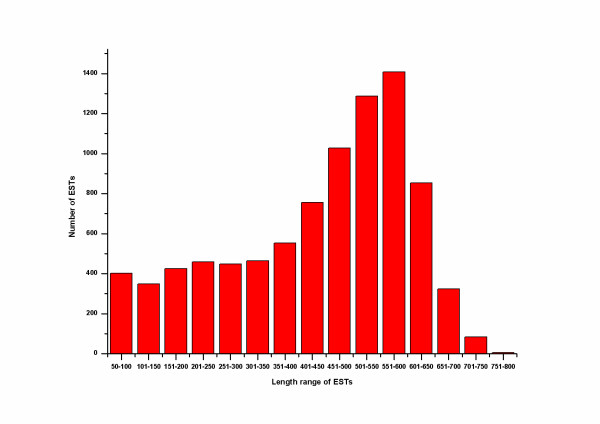
**The distribution of the reads length of the ESTs**. A total of 8696 ESTs were generated in the present study. Abscissa (50 bp) is the length of sequences, while the *Y-*coordinate is the number of ESTs. A large fraction of ESTs is between 350 and 650 bp.

**Figure 2:**
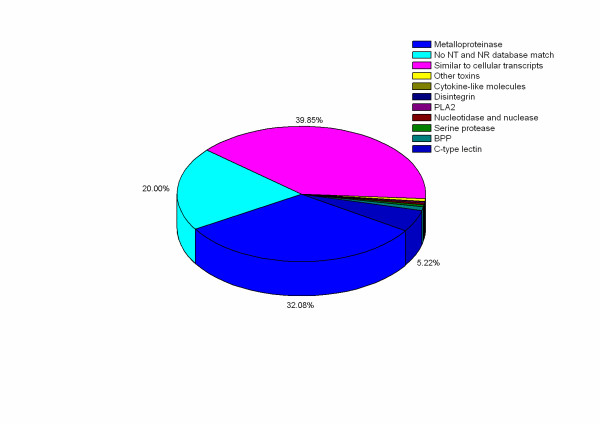
**Distribution of ESTs from *D. acutus *venomous gland according to their cDNA products**. The percentage of ESTs classification more than 1.00% is indicated.

**Figure 3:**
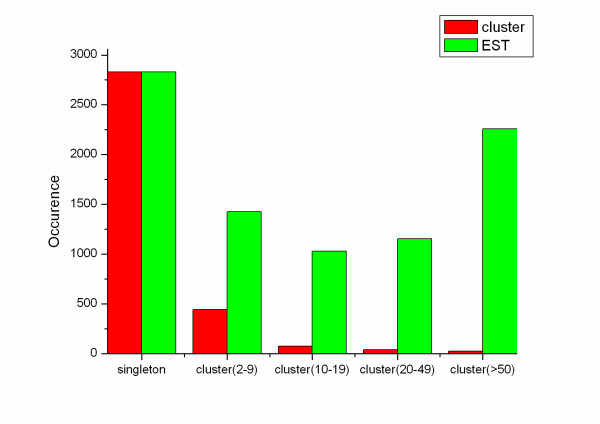
**Prevalence distribution of the cluster size**. The initial 8696 ESTs were grouped into 3416 clusters, consisting of 25 clusters (2257 of 8696 ESTs) that compromised of more than 50 ESTs each cluster, 39 clusters contained 20~49 ESTs (1153 of 8696 ESTs), 75 clusters contained 10~19 ESTs (1029 of 8696 ESTs), 445 clusters comprised 2~9 ESTseach cluster (1425 of 8696 ESTs), and 2832 unique sequence.

After discarding the poor-quality sequences, 8696 high-quality ESTs were used to analyze gene expression profile in the venom gland of *D. acutus*. The mean read length of ESTs was 398 bp (ranging from 50 bp to 772 bp, Figure 1). Subsequently, ESTs were clustered into 3416 clusters, of which 118 clusters (40.16% ESTs) associated with toxin function has been reported elsewhere [11]. In this report, we discussed mainly 1184 clusters (39.85% ESTs) involved in the cellular functional transcripts and other novel sequences (Figure 2). The distribution of all ESTs was followed:

(1) Twenty-five clusters consisted of more than 50 ESTs each, which represented the most abundant transcripts and encoded known proteins. They constituted 0.73% of the total clusters (25 of 3416 clusters) including 26.16% of total ESTs (2275 of 8696 ESTs). Interestingly, half of the most abundant transcripts were previously reported metalloproteinase from venom gland of *D. acutus *[11], indicating high prevalence. Of these 25 clusters, eight clusters are known genes that belong to housekeeping genes and two are toxin secretion related genes (Table 1).

(2) Thirty-nine clusters consisted of 20–49 ESTs each and represented 1.14% of the total clusters (39 of 3416 clusters) and 13.26% of the total ESTs (1153 of 8696 ESTs). Of 39 clusters, 13 encoded non-toxin functional proteins, such as myosin, NADH dehydrogenase subunit, cytochrome oxidase subunit and calmodulin protein. They are the second most abundant mRNA transcripts in the venom gland of *D. acutus*.

(3) Seventy-five clusters contained 10–19 ESTs each, and represented 2.20% of the total clusters (75 of 3416 clusters) and 11.83% of ESTs (1029 of 8696 ESTs), of which 17 clusters encoded the genes for troponin, ATPase subunits, retrotransposable-like elements and elongation factor 1-alpha, etc. They are considered medium-sized clusters with relatively low prevalence.

(4) The low abundant 445 clusters consisted of 2–9 ESTs each and constituted 13.03% of the total clusters (445 of 3416 clusters) and 13.69% of the total ESTs (1425 of 8696 ESTs). They included many toxin coding genes, cellular functional transcripts and partial unknown protein e.g., jerdonitin, proline dehydrogenase and hypothetical proteins.

(5) There are 2832 unique ESTs representing 82.9% of the total clusters (2832 of 3416 clusters) and 32.57% of ESTs (2832 of 8696 ESTs). The occurrence rate of these clusters is only once in current sequenced ESTs. They included cytokine-like molecule, zinc finger proteins, transport proteins and transcripts without hits to GenBank non-redundant proteins (nr) and nucleic acids databases (nt). The distribution of these cluster sizes are shown in Figure 3.

The cDNA library constructed is a non-normalized primary library without amplification, so the clone abundance or the cluster size presents the relative mRNA population [12]. About one-third of the total clones are singletons, and approximately one-fourth of the ESTs fit in clusters that are comprised of more than 50 ESTs, representing the complexity and specificity of the transcript population of the venom gland of *D. acutus*.

### ESTs relevant to protein processing

A homologue of *Bothrops insularis *calglandulin EF-hand protein family is identified at high abundance (81 ESTs) in current library (Table 1). Calglandulin EF-hand protein as a venom gland specific gene has been reported [13]. It has several conserved Ca^2+ ^motifs and is expressed exclusively in snake venom glands from many species, but not secreted to the venom. This protein family functions in the process of exporting toxins out of the cell and into the venom [14], implying that it plays a fundamental role in toxin secretion process [15]. In this library, three EF-hand protein families were found, of which two showed high identity with *Bothrops insularis *EF-hand protein family and another showed homology only with *Mus musculus *calmodulin. The diversity of EF-hand proteins in the venom gland of *D. acutus *may suggest the complexity of toxin secretion activity. The other high expression gene encodes the protein disulfide isomerase (PDI), which was represented by 58 ESTs in the library. The PDI from *D. acutus *showed 77.9% of identity with *Gallus gallus *PDI. The PDI is a redox protein responsible for disulfide bond assembly in the endoplasmatic reticulum. We also found a significant frequency difference of cysteine residues between toxin protein and cellular functional proteins (data not shown) in the venom gland of *D. acutus*, suggesting that the PDI plays a key role in toxin protein folding. Furthermore, heat shock proteins (HSPs) are also identified (15 ESTs) in this library including HSP20, HSP70 and HSP90. HSPs are chaperon for protein refolding and degeneration, which is possibly important to toxin proteins regeneration. There are many ribosomal proteins found in this library, which contributes to the high level of protein synthesis events. A large number of ribosomal proteins therefore are needed for the toxins synthesis [16]. Several other identified transcripts can also shed light on the physiological aspects of the venom gland secretion style. For instance, various clusters involved in transporter activity are found, e.g., ion transporters (uni4929105), nucleoside transporters (uni7320865) (see Additional File). All these suggested that the venom gland of *D. acutus *is a highly specialized active organ that plays a central role in secreting toxins and polypeptides with powerful synthesis capabilities.

### ESTs relevant to structural components and energy supply

There are abundant structural component transcripts expressed in the venom gland of *D. acutus*, encoding actin, troponin, calsequestrin and myosin (Table 1). Interestingly, these cellular structural components from the venom gland are similar to ones from mammalian muscle tissue, which may indicate that the structure of the venom gland cavity is similar to muscle tissue and contributes to the venom gland contractile activity. Accordingly, it could be explained that creatine kinase expressed highly in the venom gland of *D. acutus*, accounting for 2.49% (217 reads) of all ESTs (Table 1). Because creatine kinase is an important enzyme regulator of high-energy phosphate production and utilization within contractile tissues, high expression of the enzyme is adapted to energy need for gland contraction. Furthermore, abundant transcripts expressed in current library also involved in cytochrome b, cytochrome oxidase and NADH dehydrogenase, which are also needed to meet energy needs for toxin protein synthesis and gland contraction.

### Enzymes relevant to metabolism pathway

In this library, several enzymes in metabolic pathways such as glucose metabolism and nicotinate and nicotinamide metabolism were found (Table 2). In energy and material metabolism, 22 clusters sequences were identified to play a role in glucose metabolism. We also identified that unigene uni4505467 and unigene uni41055552 code for the 5'-nucleotidase, which suggests that *D. acutus *may possess a functional pathway for purine metabolism and nicotinate and nicotinamide metabolism in the venom gland. The 5'-nucleotidase participates not only in purine metabolism but also in nicotinate and nicotinamide metabolism. Snake envenomation employs three well-integrated strategies: prey immobilization via hypotension, prey immobilization via paralysis, and prey digestion. Purines (adenosine, guanosine and inosine) constitute the perfect multifunctional toxins, and evidently play a central role in all three envenomation strategies of most advanced snakes [17].

### ESTs relevant to other function

Surprisingly, 18 clusters (21 ESTs) encoding for reverse transcriptase were found in current library. They are similar to reverse transcriptase from teleost LINE family SW1 [18]. At the same time, we identified retrotransposable-like elements in this library (16 ESTs), most of them similar to ORF2 protein from a *Platemys spixii *retrotransposon CR1 [19]. So we could expect an intact retrotransposable structure in the *D. acutus *genome. This specific retrotransposable element in the *D. acutus *genome may be adapted to environmental diversity and prey need.

**Figure 4:**
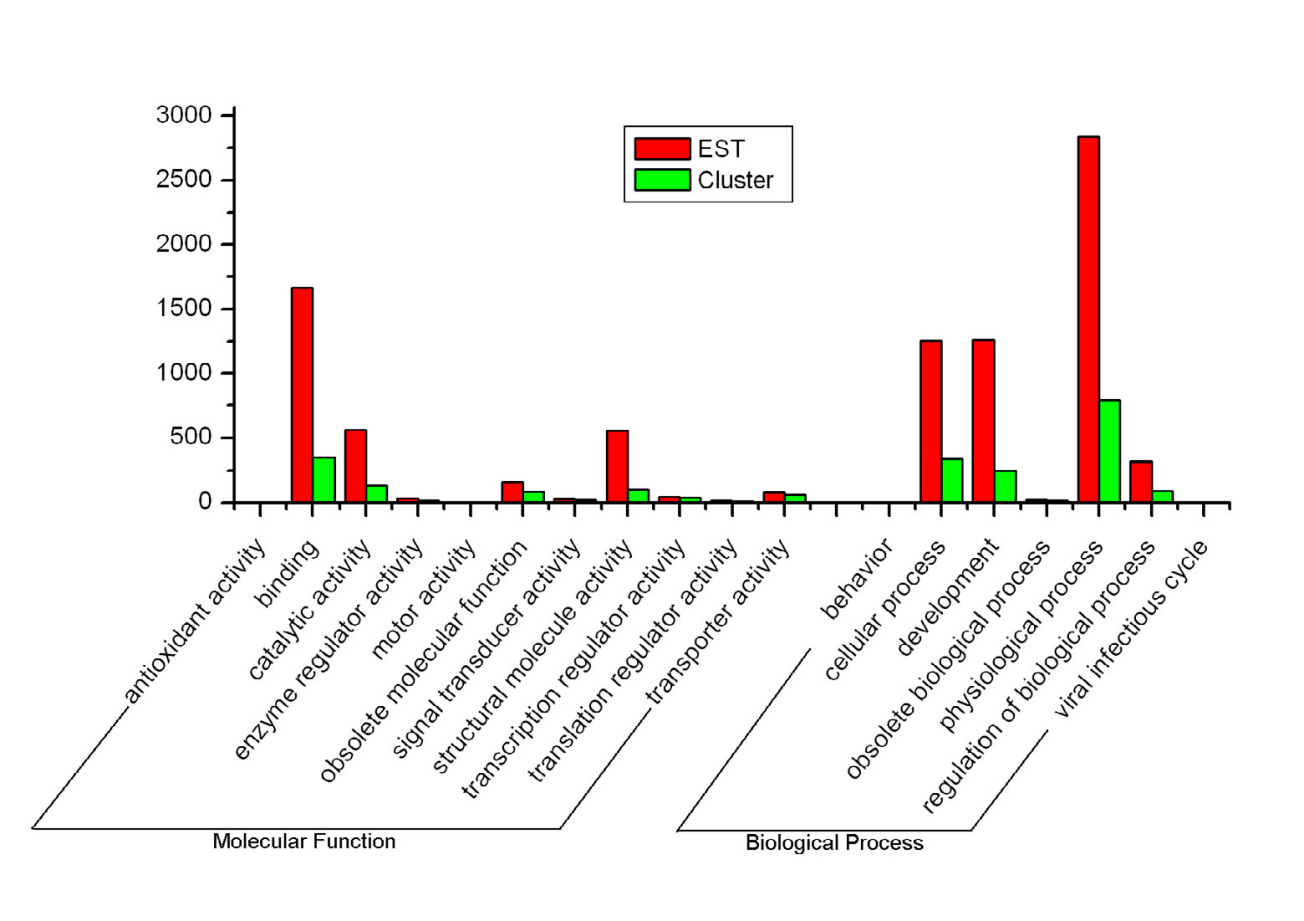
**Functional classification of transcripts in the venom gland of *D. acutus *according to molecular function and biological process based on Gene Ontology**. The vertical axis shows the number of EST or cluster.

So far, we still have not determined the complete functional categories of genes expressed in the venom gland of snake. To give an overview of the major cellular roles, the number of partial mRNA transcripts represented in each category is listed (Additional file) based on molecular function of Gene Ontology [20]. A major proportion (38 clusters) represent transcripts involved in the binding category, corresponding to 34.86% of genes of molecular function and 1.33% of total unigenes, respectively. Based on Gene Ontology function classification, 824 clusters (3144 ESTs) are assigned into the organizing principle of molecular function and 1719 clusters (5472 ESTs) of biological process (Figure 4). However, such an analysis only gave a hint of what the function might be and, in many cases, extensive biochemical and biological work is necessary to unambiguously identify a gene and its function [21]. We have presented here an initial analysis of those relevant to physiological cellular proteins.

### ESTs identifying no significant matches to known genes

There are 1553 clusters (54.40%) without significant homology to any known genes in GenBank. According to sequences discarding criteria (less than 50 bp), we could exclude the possibility that too short sequences lead to no hits. The high abundant novel sequences represent a large number of unidentified genes, suggesting the complexity and diversity of genes expressed in the venom gland of *D. acutus*. In addition, among those clusters without significant matches to known genes, 11 clusters have matches with the dbEST database and 344 clusters with the hmmpfam database. Some of those clusters, such as unigene rfstca0_000120.y1.scf showed a putative toxin-related motif region of disintegrin, and unigene rfstda0_001953.y1.scf for Conotoxin I-superfamily, indicated new toxins among those sequences. The high abundance of these sequences might correspond to the unknown toxin genes stored in the venom gland of *D. acutus*. Further study of these novel genes expressed in this specialized organ could disclose the mechanism of toxin secretion and the evolution of the snake venom gland.

### Comparative analysis with other snakes venom glands

Although several cDNA libraries from the venomous gland of a few snakes have been reported and characterized [10,15,22], analysis of transcripts from these cDNA libraries seldom involve cellular functions as the main attention was focused on the toxin components. Many components of toxin in the venom gland have been identified [11], but nerve growth factor (NGF) has not been identified in this library. In contrast to previous reports [10,15], we postulate an alternative possibility for not identifying NGF in this library: one is that the NGF might express under the specifically physiological conditions, such as in milking venom gland of *D. acutus*; another is that NGF is not a necessary component of toxin of *D. acutus*. Furthermore, a lot of clusters that may be involved in many physiological process of venom gland remained to be deciphered. It is significant to study the gene expression of venom gland of snake on cellular structure and functional aspects, which will improve the study of some physiological process such as organogenesis, cell differentiation and protein synthesis. Alternatively, some secreted membrane proteins may represent antigens of potential importance to immune control.

### Phylogenetic analysis of toxin related genes in *D. acutus*

Snake venom glands evolved once, at the base of the colubroid radiation, 60–80 million years ago, with extensive subsequent "evolutionary tinkering" [23,24]. The advanced snakes (superfamily *Colubroidea*) make up >80% of about 2900 species of snake currently described, and contain all of the known venomous forms [1]. Generally in this library, toxin clusters match sequences from snake sources in database while the cellular functional transcripts are identified by its similarity to model organisms such as *Gallus gallus, Homo sapiens *and *Rattus norvegicus*. Of note, although those transcripts of toxin are always phylogenetically closer from another snake, the average of similarities over toxins and cellular functional transcripts has no obvious difference. From the view of evolution, we postulated that the toxins tend to diverge due to natural selective pressure to adapt to different environmental conditions (mainly distinct preys). Whereas, most of the cellular components showing similarities with mammalian proteins, although they are usually phylogenetically distant, correspond to proteins of conserved functions among vertebrates and thus show higher homology [15].

**Figure 5:**
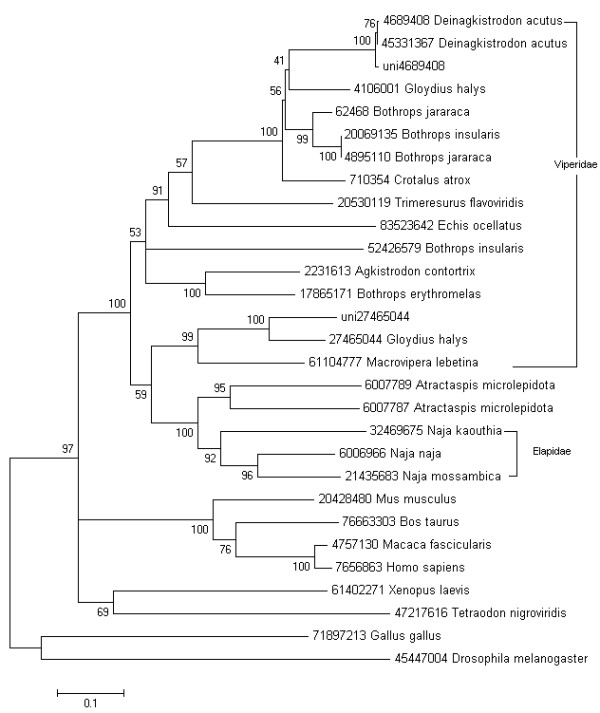
**Molecular phylogenetic analysis of the group III metalloprotease using Neighbor-joining method**. To minimize confusion, all proteins sequences from database are referred to their NCBI accession numbers. The sequences from the current library are indicated with prefix "uni".

**Figure 6:**
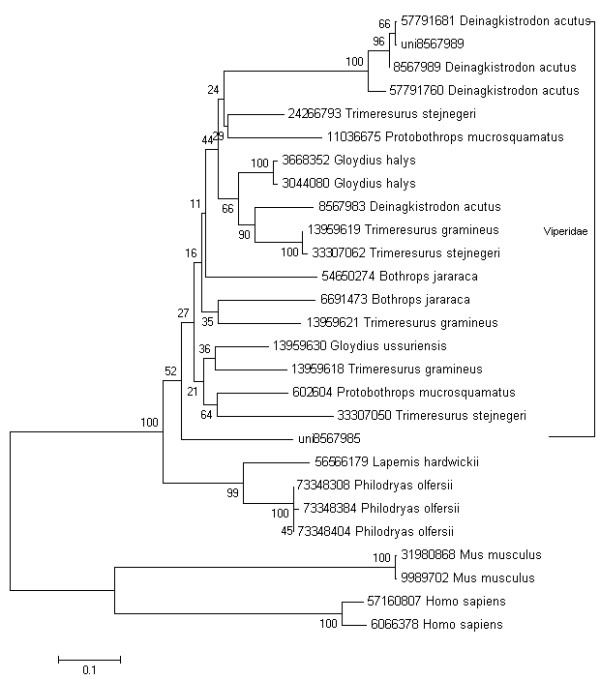
**Molecular phylogenetic analysis of the serine proteinase using Neighbor-joining method**. To minimize confusion, all proteins sequences from database are referred to their NCBI accession numbers. The sequences from the current library are indicated with prefix "uni".

The origin and evolution of many toxin gene families in the advanced snakes have been researched extensively [1,24,25]. Among these toxin gene families, most were recruited into the advanced snakes before the split of elapids and viperids. However, the phylogenetic analysis of a few other toxin gene families, such as phospholipase A2 and natriuretic toxins provide a clear evidence of an independent recruitment event. Because of limited toxin gene sequences in public databases, the origin and evolution of a number of toxin gene families remain unknown. In this report, we analyzed the phylogeny of the group III snake venom metalloproteinase and serine protease. The group III snake venom metalloproteinase consists of a proprotein, a metalloproteinase, a disintegrin and a cysteine-rich domain. It inhibits the integrin receptor selectively. Figure 5 described the phylogenetic analysis of this group III metalloproteinase in Colubroid. The phylogenetic model of this metalloproteinase is similar to CRISP protein [24], which was recruited into the advanced snakes before the split of elapids and viperids. That is the advanced snake acquired the group III metalloproteinase genes by an early single recruitment event. Subsequently, this metalloproteinase family evolved independently in elapids and viperids. We also analyzed the phylogeny of the serine proteases in suborder *Colubroidea *and similar results were shown (Figure 6). But serine protease genes have not been identified in elapids, which suggested the gene loss events in the evolutionary process or insufficient sequences information of elapids.

## Conclusion

This study identified and characterized the cellular functional transcripts in the venom gland of *D. acutus *extensively. Our aim is to develop a catalog of genes transcribed in this snake venom gland. We hoped to discover as many toxin and cellular coding genes as possible by constructing five fraction sub-libraries. The venom gland of *D. acutus *expresses many protein-coding sequences that are too divergent from those at present in GenBank to provide identification. Moreover, we found as many unidentified protein-coding sequences as identified ones. The prevalence distribution also provides a reasonable estimation of the actual frequency of these sequences in the venom gland of *D. acutus*. Furthermore, we have described a number of recognized molecules previously not known to be expressed in the venom gland of *D. acutus*, i.e., dehydrogenase and calglandulin EF-hand protein families. Though it is of relatively small size, analysis of this set of ESTs has yielded several kinds of useful information pertaining to the unmilked venom gland of *D. acutus*. It is, therefore, likely that the generation of more sequence data will result in the identification of novel *D. acutus *genes. In addition, many ESTs do not have significant database matches and these open up new avenues of exploration.

This report successfully provides evidence about the function of snake venom gland and a source of reptilian sequences. Gene cataloguing and profiling of the venom gland of *D. acutus *is an essential requisite to provide molecular reagents for functional genomic studies needed for elucidating mechanisms of action of toxins and surveying physiological events taking place in the very specialized secretory tissues. So this study provides a first global view of the genetic programs for the venom gland of *D. acutus *described so far and an insight into molecular mechanism of toxin secreting in the very specialized organ.

## Materials and methods

### Library construction

A non-normalized cDNA library was generated from the venom gland of *D. acutus*. The *D. acutus *was captured from Wuyi Mountain, Fujian Province, China. After killing the snake by cutting the head, the venom glands were recovered. All tissues were immediately frozen in liquid nitrogen. The total RNA was isolated with Trizol Reagent (Invitrogen) and the mRNA was purified with Oligotex mRNA Kits (Qiagen). The cDNA synthesized with Superscipt II-RT (Invitrogen) and DNA polymerase I (Promega), was flanked by EcoR I adaptor (Stratagene) and treated by Xho I (Stratagene). The double strand cDNA was extracted by electrophoresis separation with five fractions (<0.25 kb, 0.25~0.5 kb, 0.5~1 kb, 1~2 kb and > 2 kb) and then cloned into EcoR I and Xho I digested pBluescript II vector. The plasmid was transformed into *E. coli *(DH10B) to amplify the cDNA.

### EST sequencing, data processing and bioinformatics analysis

The cDNA clones were picked randomly. Plasmids were isolated according to a standard alkaline lysis protocol, and sequenced with MegaBACE 1000 (Amersham Pharmacia). Base-Calling was performed with PHRED [26], the cutoff Phred score was 20 [27]. Original sequences were generated by removing vector and *E. coli *DNA sequences using Cross-match [26]. High-quality ESTs were assembled into contigs using Phrap software [26]. Default settings were used except 40 bp minimum overlap and 99% identity. To assign annotation to the assembled ESTs (clusters), these sequences were searched against the nr (E values < 1e-5) and nt (E values < 1e-10) for homology comparison using BLASTX and BLASTN [28]. Then those clusters without significant matches to known genes were compared to the dbEST database by BLAST and pfam database by hmmpfam [29]. All assembled sequences having the same annotation were further clustered into a unique gene. Based on Gene Ontology classification, we constructed a gene expression profile of the venom gland of *D. acutus*. In order to comprehend the role of those transcripts played in the biochemical process, we assigned enzyme functions and enzyme commission numbers to reconstruct anabolic and catabolic pathways through linkage to biochemical pathways in the KEGG database [30]. The phylogenetic analysis of toxin protein families was conducted by MEGA 3.1 [31].

## Authors' contributions

ZB, LQ participated in the design of study, carried out the experiments and the comparative analysis, and drafted the manuscript. YW participated in the design of study. ZX, LY participated in the design of study and the data analysis. HY, QP, SX participated in the data analysis. YJ, HS, YG conceived the study and participated in coordinate and help to draft the manuscript. All authors have read and approved the paper.

## Figures and Tables

**Table 1:** Identification of the high abundance cluster (≥10 reads) of cellular functional transcripts

Cluster^a^	reads^b^	E-value	Annotation^c^
uni8571478	217	0	Creatine kinase [*Zaocys dhumnades*]
uni33563240	190	0	Actin, alpha 1, skeletal muscle [*Mus musculus*]
uni5359759	124	1.00E-65	Fast troponin T isoform [*Coturnix japonica*]
uni23506661	81	1.00E-78	Calglandulin EF-hand protein [*Bothrops insularis*]
uni6978487	79	0	Aldolase A [*Rattus norvegicus*]
uni64277	67	1.00E-170	Calsequestrin [*Rana esculenta*]
uni131109	65	5.00E-51	Parvalbumin beta
uni3582124	59	0	Cytochrome oxidase subunit I [*Dinodon semicarinatus*]
uni21703694	58	0	Protein disulfide isomerase [*Gallus gallus*]
uni45382253	53	7.00E-79	Troponin I [*Gallus gallus*]
uni23480590	48	2.00E-10	Hypothetical protein [*Plasmodium yoelii yoelii*]
uni6708502	47	0	Superfast myosin heavy chain [*Felis catus*]
uni18378003	40	1.00E-95	NADH dehydrogenase subunit 1 [*Homoroselaps lacteus*]
uni50845428	29	8.00E-76	Atrial/embryonic alkali myosin light chain [*Homo sapiens*]
uni12004999	29	2.00E-42	Fast skeletal muscle troponin T isoform 2-e16 [*Mitu tomentosa*]
uni3582128	28	1.00E-101	Cytochrome oxidase subunit III [*Dinodon semicarinatus*]
uni136089	28	1.00E-126	Tropomyosin beta chain (Tropomyosin 2) (Beta-tropomyosin)
uni15011037	24	1.00E-137	cytochrome b [*Crotalus horridus atricaudatus*]
uni50513993	22	2.00E-74	Chain B, Crystal Structure Of The Sr Ca2+-Atpase With Bound Amppcp
uni104550	21	0	Ca2+-transporting ATPase
uni46048961	21	1.00E-176	Glyceraldehyde-3-phosphate dehydrogenase [*Gallus gallus*]
uni168777	20	4.00E-12	Calmodulin-like 3 [*Mus musculus*]
uni31981562	20	1.00E-136	Pyruvate kinase 3 [*Mus musculus*]
uni52078482	19	4.00E-92	Myosin light chain [*Oxyuranus scutellatus scutellatus*]
uni45384350	15	4.00E-25	60S acidic ribosomal protein P1 [*Gallus gallus*]
uni51460955	15	8.00E-96	PREDICTED: similar to CAVP-target protein (CAVPT) [*Homo sapiens*]
uni45384494	14	1.00E-101	Acidic ribosomal phosphoprotein [*Gallus gallus*]
uni3582127	14	6.00E-59	ATPase subunit 6 [*Dinodon semicarinatus*]
uni3582125	14	2.00E-87	Cytochrome oxidase subunit II [*Dinodon semicarinatus*]
uni3582131	14	1.00E-107	NADH dehydrogenase subunit 4 [*Dinodon semicarinatus*]
uni47212235	14	1.00E-114	Unnamed protein product [*Tetraodon nigroviridis*]
uni119133	13	0	Elongation factor 1-alpha (EF-1-alpha)
uni28277353	13	7.00E-32	Eno3-prov protein [*Xenopus laevis*]
uni86460	13	6.00E-60	Troponin T, skeletal muscle, isoform 1 – chicken
uni33086612	12	9.00E-48	Aa1-330 [*Rattus norvegicus*]
uni33086544	12	2.00E-59	Ab2-057 [*Rattus norvegicus*]
uni33694242	12	1.00E-100	Ribosomal protein L15 [*Homo sapiens*]
uni45382453	11	1.00E-107	Elongation factor 2 [*Gallus gallus*]
uni6576738	10	6.00E-98	ORF2 [*Platemys spixii*]
uni45382061	10	1.00E-129	Triosephosphate isomerase (TIM, D-glyceraldehyde 3-phosphate ketol-isomerase) [*Gallus gallus*]

^a ^Unigenes were obtained using Phrap software and clustered manually.

^b ^Number of sequenced clones in unigene.

^c ^The best match from BLASTX.

**Table 2:** Proteins from venom gland of *D. acutus *predicted to play a role in physiological metabolism pathway

Unigene	EC number	Enzyme name and synonyms
Glucose metabolism		
Uni1351884	1.1.1.1	Alcohol dehydrogenase, zinc-containing
Uni34555782	1.1.1.27	L-lactate dehydrogenase
Uni17369829	1.1.1.27	L-lactate dehydrogenase
Uni46048961	1.2.1.12	Glyceraldehyde 3-phosphate dehydrogenase
Uni50754481	1.2.4.1	Pyruvate dehydrogenase complex, E1 component, alpha subunit
Uni50760204	2.3.1.12	Acetoin dehydrogenase complex, E2 component, dihydrolipoamide acetyltransferase, putative
Uni45383682	2.7.1.11	6-phosphofructokinase
Uni206205	2.7.1.40	Pyruvate kinase
Uni31981562	2.7.1.40	Pyruvate kinase
Uni45384486	2.7.2.3	Phosphoglycerate kinase
Uni51950293	3.1.3.13	Unknown
Uni50593010	3.1.3.13	Unknown
Uni34867424	3.6.1.7	Putative acylphosphatase
Uni6978487	4.1.2.13	Fructose-bisphosphate aldolase
Uni18307578	4.1.2.13	Fructose-bisphosphate aldolase
Uni28277353	4.2.1.11	Enolase
Uni20067631	5.3.1.9	Glucose-6-phosphate isomerase
Uni51950293	5.4.2.1	Similar to 2,3-diphosphoglycerate-dependent phosphoglycerate mutase
Uni50593010	5.4.2.1	Similar to 2,3-diphosphoglycerate-dependent phosphoglycerate mutase
Uni51950293	5.4.2.4	Unknown
Uni50593010	5.4.2.4	Unknown
Uni47224301	6.2.1.1	Acetyl-CoA synthetase
		
Purine metabolism		
Uni41393093	1.1.1.205	Similar to IMP dehydrogenase
Uni53129754	1.1.1.205	Similar to IMP dehydrogenase
Uni51513483	2.4.2.7	Adenine phosphoribosyltransferase
Uni601873	2.4.2.8	Hypoxanthine phosphoribosyltransferase
Uni206205	2.7.1.40	Pyruvate kinase
Uni31981562	2.7.1.40	Pyruvate kinase
Uni50745642	2.7.6.1	Ribose-phosphate pyrophosphokinase
Uni28278826	2.7.7.6	DNA-directed RNA polymerase, beta subunit
Uni34873368	2.7.7.6	DNA-directed RNA polymerase, beta subunit
Uni18032797	2.7.7.6	DNA-directed RNA polymerase, beta subunit
Uni5739210	2.7.7.7	DNA polymerase III, epsilon subunit
Uni41055552	3.1.3.5	5'-nucleotidase
Uni4505467	3.1.3.5	5'-nucleotidase
Uni50731966	4.6.1.1	Putative adenylate/guanylate cyclase transmembrane protein
		
Nicotinate and nicotinamide metabolism		
Uni13489054	2.7.1.-	Probable serine/threonine-protein kinase
Uni4507831	2.7.1.-	Probable serine/threonine-protein kinase
Uni45384254	2.7.1.-	Probable serine/threonine-protein kinase
Uni50761892	2.7.1.-	Probable serine/threonine-protein kinase
Uni50752935	2.7.1.-	Probable serine/threonine-protein kinase
Uni7710088	2.7.1.-	Probable serine/threonine-protein kinase
Uni41055552	3.1.3.5	5'-nucleotidase
Uni4505467	3.1.3.5	5'-nucleotidase
Uni34852937	3.5.1.-	UDP-3-0-acyl N-acetylglucosamine deacetylase
